# Gut Melatonin in Vertebrates: Chronobiology and Physiology

**DOI:** 10.3389/fendo.2015.00112

**Published:** 2015-07-22

**Authors:** Sourav Mukherjee, Saumen Kumar Maitra

**Affiliations:** ^1^Department of Zoology, Visva-Bharati University, Santiniketan, India

**Keywords:** gut melatonin, AANAT, chronobiology, physiology

## Abstract

Melatonin, following discovery in the bovine pineal gland, has been detected in several extra-pineal sources including gastrointestinal tract or gut. Arylalkylamine *N*-acetyltransferase (AANAT) is the key regulator of its biosynthesis. Melatonin in pineal is rhythmically produced with a nocturnal peak in synchronization with environmental light–dark cycle. A recent study on carp reported first that melatonin levels and intensity of a ~23 kDa AANAT protein in each gut segment also exhibit significant daily variations but, unlike pineal, show a peak at midday in all seasons. Extensive experimental studies ruled out direct role of light–dark conditions in determining temporal pattern of gut melatoninergic system in carp, and opened up possible role of environmental non-photic cue(s) as its synchronizer. Based on mammalian findings, physiological significance of gut-derived melatonin also appears unique because its actions at local levels sharing paracrine and/or autocrine functions have been emphasized. The purpose of this mini review is to summarize the existing data on the chronobiology and physiology of gut melatonin and to emphasize their relation with the same hormone derived in the pineal in vertebrates including fish.

## Introduction

Extensive research carried out in past 50 years have clearly depicted melatonin (5-methoxy-*N*-acetyltryptamine) as a potent chronobiotic molecule involved in the regulation of a variety of physiological functions (1). Following discovery in the bovine pineal gland (2), melatonin is detected in several non-pineal tissues/organs, such as retina, Harderian gland, and gastrointestinal tract (GIT) or gut. However, existing knowledge on melatonin has stemmed largely from the studies on pineal that too in mammals, leaving non-pineal tissues, especially in lower vertebrates, as an interesting topic of research. Though functional characterization of melatonin in gut, relative to that in retina and Harderian gland, has received serious attention, the studies are limited mostly to the mammals, especially rodents and pigs. Moreover, current knowledge on the cellular localization, distribution, and temporal pattern of gut melatonin is scarce and inconsistent as well. Thus, a brief review of the existing literature on gut melatonin appears meaningful for understanding its unique features in vertebrates.

## Cellular Localization and Distribution

Localization of melatonin in the enterochromaffin cells (EC) of digestive mucosa of rat was followed by its quantitative estimation in gut tissues. At the sub-cellular level, strongest binding was noted in nuclear fraction, followed by microsomal, mitochondrial, and cytosolic fractions (3). In mammals, melatonin-producing cells were found in submucosa and muscularis layer of esophagus, the glandular portion of the gastric wall and in the area of Lieberkühn’s crypts and Brunner’s glands of duodenum, and more specifically in EC of mucosal layer (3). As in birds and mammals, maximum immunoreactivity was noted at the outer margin of lamina propria in mucosal layer of intestinal villi in carp gut (4).

Generally, melatonin concentrations in gut tissues surpass the levels of melatonin in circulation by 10–100 times (3). Daytime levels of gut melatonin were measured in several species (5) of fish, e.g., sturgeon, rainbow trout, and carp (stomach ~102 pg/g, proximal gut or PG ~146 pg/g, and distal gut or DG ~141 pg/g); amphibians, e.g., axolotl (stomach and PG ~44 pg/g and DG ~92 pg/g), and bullfrog (esophagus ~73 pg/g, stomach ~78 pg/g, PG ~20 pg/g, and DG ~152 pg/g); and reptiles, e.g., red-sided garter snake (stomach ~1018 pg/g, PG ~328 pg/g, and DG ~511 pg/g). The midday values of melatonin in the anterior (~550 pg/g), middle (~538 pg/g), and posterior (~578 pg/g) segments of gut in a day-active carp (6) did not indicate any regional variations in its distribution in the same animal, but depicted species-specific variations in a particular gut segment (5). However, it remains obscure whether gut melatonin levels vary between the day-active and night-active animals.

## Biosynthesis of Melatonin

The synthesis of melatonin in all the melatonin-synthesizing cells is a four-step phenomenon. First, the precursor l-tryptophan is taken up from the circulation (blood) and is converted to 5-hydroxy-tryptophan (5-HTP) in the mitochondria by Trp-5-mono-oxygenase/hydroxylase and is then decarboxylated in the cytosol by l-aromatic amino acid decarboxylase to form serotonin (5-hydroxytryptamine, 5-HT), that in turn is acetylated (*N*-acetylation) into *N*-acetyl serotonin by arylalkylamine-*N*-acetyltransferase (AANAT) (7), which is considered as the rate-limiting enzyme in melatonin biosynthetic pathway. Finally, *N*-acetyl serotonin is O-methylated by hydroxyindole-O-methyltransferase (HIOMT) to form melatonin (8).

Endogenous melatonin biosynthesis within the ECs of the digestive mucosa has been evident from the studies on the expression of genes for two key melatonin-synthesizing enzymes. The study of gut tissues detected mRNA expression of *Aanat* in rat (9) and *Hiomt* in quail (10) as well as goldfish (11). *Aanat*-2 expression, as noted in the gut of goldfish (11) and rainbow trout (12), was supported by densitometric analysis of AANAT protein in the carp gut (6). The study detected a ~23 kDa AANAT protein corresponding to AANAT in the pineal of pike, trout, and carp (13) in gut tissue homogenates and thereby ensured endogenous synthesis of melatonin in gut.

## Chronobiology

### Temporal pattern of gut melatonin

**Figure 1:**
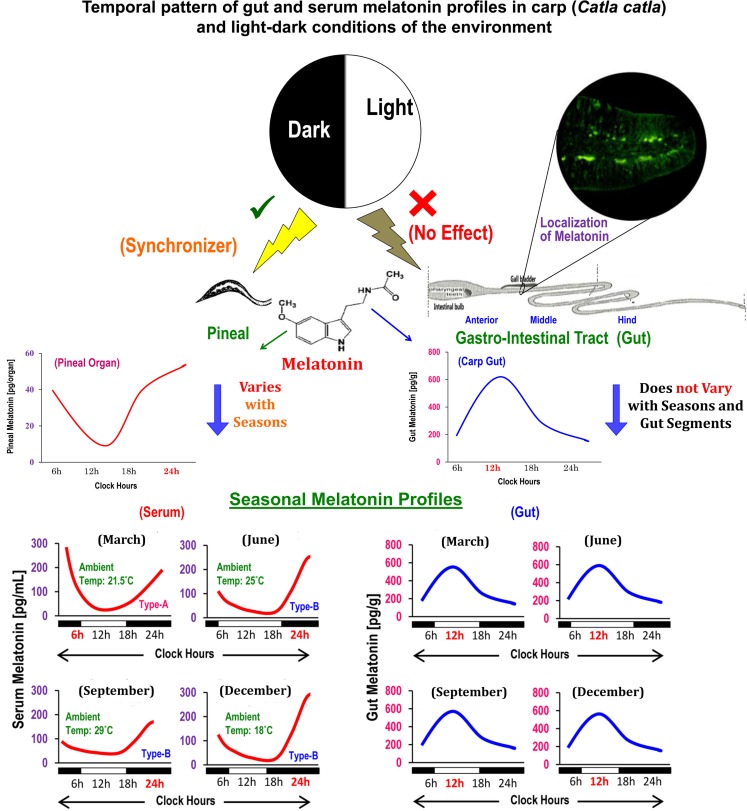
**Schematic presentation of the summary of existing information on the localization, distribution, and temporal organization of melatonin concentrations in different anatomical segments of gut in relation to the diurnal and seasonal profiles of serum melatonin in carp *Catla catla* and light–dark cycle in the environment**.

Circulating profiles of melatonin in different vertebrates (14), including carp (15), exhibit precise diurnal rhythms with a peak during the dark phase and nadir during the photo phase and such rhythms are primarily generated by pineal AANAT (7). Until recently, comparable data on the temporal pattern of gut melatonin and its regulatory mechanisms were unknown for any vertebrates. A study on mice reported lower melatonin level during the day relative to nocturnal values only in the duodenum–jejunum segment of the GIT (16). In goldfish, though titers of melatonin were not measured, analysis of *Aanat*-2 mRNA expression revealed a daily rhythm in hindgut, but not in foregut (11). Importantly, the daily peak in the *Aanat*-2 mRNA expression persisted under continuous light as well as continuous darkness (11). Conversely, the study on carp by showing parallel changes in the levels of melatonin and AANAT density for the first time demonstrated that melatonin-synthesizing system in each gut segment, irrespective of seasons, undergoes significant daily variations with a peak at midday (6). Such findings also opened up a possibility that regulatory mechanism of melatonin synthesis in gut and pineal in the same animal species (Figure 1) is different (13).

### Influence of photoperiods on rhythmicity in gut melatonin

The duration of light and thereby duration of darkness in a 24-h cycle is known to play a key role in the regulation of melatonin synthesis in the pineal (14). It is noteworthy that in fish, including carp (13), light acts directly on the pineal gland. By contrast, in mammals, light is detected by the retina and affects the pineal gland indirectly with a multisynaptic pathway (14). However, the question whether lighting conditions play a critical role of a synchronizer or regulator of gut melatonin was not duly addressed. Thus, a study was undertaken with carp (17), which due to its natural surface dwelling habit maintains close contact with environmental light and thereby is considered as an ideal model for the study of photo-response mechanisms in any melatonin-synthesizing tissue. Accordingly, the carp were held either under altered long (LP) or short photoperiods (SP) in a 24-h cycle, or under continuous light (LL) or darkness (DD) for equal duration. The results of the study indicated that none of the employed photo-regimens has any significant effect *per se* on the daily profiles of gut melatonin levels and AANAT protein density (17). Notably, an earlier study on the same carp demonstrated that diurnal rhythmicity in serum melatonin and pineal AANAT with a nocturnal peak (13) was lost when fish were held under LL or DD. Taken together, it is suggested that melatonin synthesis in gut, unlike pineal, is not a dark-dependent phenomenon, and environmental lighting conditions may not serve the role of external cue(s) in determining its rhythmic features in a daily cycle.

### Influences of food on rhythmicity in gut melatonin

One of the periodic variables in the gut is the availability of food, which may serve as an important cue to determine daily periodicity of melatonin synthesis in gut (18). Notably, the study on the distribution of melatonin in different parts of the GIT in cow (poly-gastric) and pig (mono-gastric) revealed that cows had higher melatonin levels in stomach and ileum, but lower in cecum and colon (19). Accordingly, a relation between melatonin secretion and the digestive functions is sought. Depending on the meal frequency and timing of meals, several circulating metabolites and hormones undergo daily variations (20), and these variations are dependent on whether the animal is fed, fasted, or starved. Because timing of food intake is roughly opposite in phase between diurnal and nocturnal species, it is likely that synthesis of gut melatonin is correlated with feeding in various animals including fish species. However, any data from appropriate experimental studies that could support the hypothesis on a direct role of food availability and its timings on gut melatoninergic system in any animal species still remain unknown.

## Physiology of Gut Melatonin

Melatonin is a lipophilic compound diffusing rapidly through biological membranes and actions in an endocrine and/or, paracrine and/or, autocrine manners. It is involved in the regulation of multiple functions, including the control of gastrointestinal system. Melatonin produced by EC may perform paracrine functions, while its action in the intestinal muscles may be either direct or it may act via the myenteric nervous system (3, 19).

A study on rainbow trout demonstrated that melatonin is released from gastrointestinal tissue and addition of l-tryptophan to the incubation medium stimulates melatonin synthesis and release (21). The presence of melatonin in intestinal villi indicated that melatonin might be involved in the transmembrane transport of electrolytes and ions. Moreover, melatonin increased water content in feces (22) and the topical application of melatonin stimulated short-circuit current in colonic cells (23). Experimental studies confirmed that melatonin also inhibited the contraction of smooth muscles in stomach, ileum, and colon (24). In rats, endogenous melatonin affected the electro-myogram of pre- and post-prandial motility, though such actions were observed only at night (25), when the concentration of melatonin in blood remained usually high. Perhaps, melatonin relaxes gastrointestinal muscles by specifically blocking nicotinic channels (26).

The assumption that melatonin may also interact with dopamine-sensitive, possibly Ca^2+^-activated, K^+^ channels (27) earned support from the study, in which inhibition of small-conductance K^+^ channels attenuated melatonin-induced relaxation of gastric muscles (28). In addition to its relaxation effect presented in several *in vitro* studies, melatonin appeared to inhibit serotonin (5-HT) action also *in vivo*. Serotonin facilitated the “food transit time” (FTT) (the moment of food intake to the appearance of the first feces stained with food coloring), as compared to controls, whereas injection of melatonin to serotonin implanted mice significantly increased the FTT (19). In a similar study, small doses of melatonin relaxed the gut muscles and facilitated intestinal motility in rats (19). Further studies argued that a counterbalancing system exists between serotonin and melatonin which within the GIT melatonin functions as a physiological antagonist of serotonin (22). High dose of melatonin inhibited the spontaneous or serotonin-induced contraction of GIT muscles and induced intestinal elongation. Conversely, low doses of melatonin stimulated intestinal contraction, resulting in the shortening of gut (3).

Several mammalian studies indicated that melatonin may have a protective role against development of gastric ulcers (29, 30). It is proposed that the prevention of stress- or ethanol-induced gastric lesions in rats is probably due to the anti-serotoninergic effect of melatonin (29). The incidence and severity of spontaneously induced gastric ulcers are significantly reduced in pigs, which were fed with 5 mg melatonin/kg enriched food. The highest incidence of ulcers is observed in pigs with the lowest level of melatonin in their plasma and stomach tissues (30). Protection against stress-induced lesions might be due to a strong antioxidant action of melatonin and the restoration of microcirculation (31).

In addition to its antioxidant effect, melatonin action in the prevention or treatment of colitis (32) includes the stimulation of the immune system. Though direct evidence is lacking, indications are available to show that melatonin administration to rats significantly increased the number and size of Peyer’s patches, the major immune tissue of the GIT (33). Melatonin treatment may improve irritable bowel symptoms (IBS) as well (34). Thus, over the decades, melatonin has been promoted as a “magic cure” for the treatment or prevention of several physiological disorders ranging from aging to aggression, depression to hypertension, suppressed immunity to oxidative stress, insomnia to jet lag (1). However, convincing data are yet to be known to prove that such actions are also ascribed to gut-derived melatonin.

## Conclusion

The information gathered so far provides indications that environmental and neuroendocrine regulatory mechanisms of melatonin synthesis in the pineal and in the gut are different. Likewise, functional characteristics of pineal-derived and GIT-derived melatonin may not be identical, as the nature of release and function of this extra-pineal melatonin are not yet fully understood. There are reasons to argue that pineal melatonin mostly acts as an endocrine agent, whereas GIT melatonin performs not only endocrine functions but also in autocrine and paracrine manners (18). However, emergence of a general idea on the physiological significance of gut melatonin has suffered a major setback due to lack of data from studies on any non-mammalian species and thereby warrants further carefully controlled research under diverse experimental conditions using animals representing different groups of vertebrates.

## Conflict of Interest Statement

The authors declare that the research was conducted in the absence of any commercial or financial relationships that could be construed as a potential conflict of interest.
